# Pathological evaluation of neoadjuvant chemotherapy in advanced gastric cancer

**DOI:** 10.1186/s12957-018-1534-z

**Published:** 2019-01-03

**Authors:** Shen-Bao Hu, Chun-Hao Liu, Xiang Wang, Yun-Wei Dong, Lin Zhao, Hong-Feng Liu, Yue Cao, Ding-Rong Zhong, Wei Liu, Yan-Long Li, Wei-Sheng Gao, Chun-Mei Bai, Zhong-Hua Shang, Xiao-Yi Li

**Affiliations:** 1 Department of General Surgery, Peking Union Medical College Hospital, Chinese Academy of Medical Sciences & Peking Union Medical College, Beijing, 100730 China; 2 Department of Medical Oncology, Peking Union Medical College Hospital, Chinese Academy of Medical Sciences & Peking Union Medical College, Beijing, 100730 China; 3 Department of Pathology, Peking Union Medical College Hospital, Chinese Academy of Medical Sciences & Peking Union Medical College, Beijing, 100730 China; 4 Department of Radiology, Peking Union Medical College Hospital, Chinese Academy of Medical Sciences & Peking Union Medical College, Beijing, 100730 China; 5 Institute of Basic Medical Sciences, Chinese Academy of Medical Sciences, School of basic Medicine, Peking Union Medical College, Beijing, 100005 China; 6 Department of General Surgery, Second Clinical Hospital of Shanxi Medical University, Taiyuan, 030001 Shanxi China

**Keywords:** Gastric cancer, Neoadjuvant chemotherapy, Graded histological regression (GHR), Overall survival (OS)

## Abstract

**Background:**

Although pathological evaluation has been considered an effective evaluation method, some problems still exist in practice. Therefore, we explored whether there are more reasonable and practical pathological evaluation criteria for neoadjuvant chemotherapy in patients with advanced gastric cancer. Here, we aim to determine pathological judgment criteria for neoadjuvant chemotherapy in patients with advanced gastric cancer.

**Methods:**

Eighty-seven patients with cT2–4 or cN+ were enrolled in this study. Pathological factors for overall survival (OS) were investigated using univariate and multivariate analyses, and the pathological criteria for neoadjuvant chemotherapy were then determined.

**Results:**

A total of 87 patients underwent 3–4 cycles of neoadjuvant chemotherapy, with 67 (77.0%), 15 (17.2%), and 5 (5.8%) receiving Folfox6, Xelox, and SOX regimens, respectively. All patients showed different levels of graded histological regression (GHR) of the primary tumor, with a ≥ 50% regression rate of 50.6%. The univariate analysis showed that GHR ≥ 50% (*p* = 0.022), 66.7% (*p* = 0.013), and 90% (*p* = 0.028) were significantly correlated with OS. The multivariate analysis demonstrated that ypTNM (II/III) stage was significantly associated with OS compared with ypTNM (0+I) stage [HR = 3.553, 95% CI 1.886–6.617; HR = 3.576, 95% CI 1.908–6.703, respectively] and that the Lauren classification of diffuse type was also an independent risk factor for OS compared with the intestinal type (HR = 3.843, 95% CI 1.443–10.237).

**Conclusions:**

The Lauren classification and ypTNM stage after neoadjuvant chemotherapy are independent prognostic factors in advanced gastric cancer. A GHR ≥ 50%/< 50% can be used as the primary criterion for advanced gastric cancer after neoadjuvant chemotherapy to determine postoperative adjuvant chemotherapy regimens.

## Background

Gastric cancer (GC) is one of the most common malignancies worldwide and ranks fifth and third with regard to the incidence and mortality, respectively, of malignant tumors [1]. A local recurrence rate as high as 50% and a long-term survival rate of less than 30% are observed among approximately 90% of patients with advanced GC, even after radical surgery [2]. Therefore, improving the efficacy of therapy for patients with advanced GC is an important aspect for overall treatment outcomes of this disease. At present, multidisciplinary treatment including neoadjuvant chemotherapy is the standard treatment for advanced GC, and many studies have shown that this approach can improve survival compared with surgery alone [3, 4]. Indeed, neoadjuvant chemotherapy can result in downstaging, reduced intraoperative dissemination, and enhanced R0 resection rates, which all improve the prognosis. Another important role of neoadjuvant chemotherapy is to evaluate the effect of the neoadjuvant chemotherapy regimen to guide the selection of the postoperative chemotherapy approach [2]. However, the current efficacy of common chemotherapy drugs is only 49–69.7% [5–13]. Of those who undergo neoadjuvant chemotherapy, the identification of patients for whom therapy would be effective is difficult due to the lack of a uniform standard assessment, which greatly influences prognosis and the options for postoperative chemotherapy regimens. One study reported that due to their respective limitations, traditional methods of imaging assessment (e.g., computed tomography (CT), magnetic resonance imaging (MRI), ultrasound, and endoscopy) are inaccurate for the evaluation of the efficacy of neoadjuvant treatment for GC [14], which impacts selection of the therapy regimen and indications for treatment outcomes.

At present, the common accepted standard for evaluation of therapeutic efficacy is pathological examination of tumor specimens from surgical resection following neoadjuvant chemotherapy to determine graded histological regression (GHR). As the most commonly used criteria, pathological efficacy evaluation (GHR ≥ 2/3 is effective) was proposed by Japan’s Gastric Cancer Research Association (JCGC) in 1999, and histopathological regression classification of primary tumor beds (GHR ≥ 90% is effective) was proposed by Becker et al. in 2003 [15, 16]. In Becker’s 2003 study, GHR ≥ 90% was observed in only 11.1% of patients (4/36), and in a later study with an increased sample size, the proportion of patients with GHR ≥ 90% was only 21.2% (102/480) [17]. The effective rate of GHR ≥ 2/3 reported by Kurokawa et al. was 34% [18]. Based on these standards, most patients with advanced GC who undergo neoadjuvant chemotherapy are faced with a replacement chemotherapy regimen after surgery, which is a burden to clinical practice. Whether the patients who have benefited from neoadjuvant chemotherapy can be identified only by these “rigorous” pathological criteria or if more reasonable criteria can be established for easier screening and implementation of clinical decisions remains unknown; therefore, this question warrants further research.

In this retrospective study, 87 patients with GC who met the inclusion criteria were regularly followed up to assess the association of each clinicopathological feature with overall survival (OS). This approach allowed for the determination of independent predictors of OS and the effective GHR standard for neoadjuvant chemotherapy in GC.

## Methods

### Study population

We included patients with advanced GC (cT2-cT4 or cN+) whose disease was confirmed by gastroscopic biopsy and who were treated with neoadjuvant chemotherapy from April 2007 to December 2015 at Peking Union Medical College Hospital. This study included 87 patients with GC who met the inclusion criteria. Clinicopathological data (such as preoperative endoscopy, endoscopic ultrasonography, enhanced CT, positron emission tomography (PET)/CT, preoperative neoadjuvant chemotherapy cycles and programs, surgical methods, postoperative pathology, and postoperative follow-up) were analyzed.

The inclusion criteria were as follows: (1) histologically confirmed GC or gastroesophageal junction cancer; (2) advanced GC (cT2–4 or cN+) according to the AJCC 8.0 staging system, as verified by ultrasound endoscopy, enhanced CT, or PET/CT; (3) preoperative treatment with chemotherapy, followed by radical R0 resection and D2 lymph node dissection; (4) patients older than 18 years with no history of tumor-related hemorrhage, a white blood cell count > 4 × 10^9^/L, hemoglobin level > 90 mg/L, platelet count > 100 × 10^9^/L, adequate liver and kidney functional reserve, approximately normal electrocardiogram, normal heart function, no history of stomach surgery, radiotherapy or chemotherapy, and no other concurrent malignant disease; and (5) patients who provided informed consent.

The exclusion criteria were as follows: (1) no preoperative chemotherapy, (2) non-R0 resection and non-D2 lymph node dissection, (3) other concurrent oncological diseases, and (4) incomplete clinicopathological data. The study was approved by the Ethics Committee of Peking Union Medical College Hospital.

### Study assessment

Assessments were performed before and after neoadjuvant chemotherapy, and more than one of the following assessment techniques were applied to determine the clinical stage: enhanced CT, ultrasound endoscopy, or PET/CT. CT was evaluated by the same experienced radiologist.

The average course of chemotherapy was 3–4 courses, and of the patients treated, 67 (77.0%), 15 (17.2%), and 5 (5.8%) were treated with the Folfox6, Xelox, and SOX regimens, respectively. After the end of chemotherapy (3–4 weeks), patients underwent radical gastrectomy, which was performed by experienced surgeons of the same surgical team. In specimens obtained by radical surgery, tumor stage and GHR were confirmed by the same experienced pathologist (DRZ). After surgery, postoperative adjuvant chemotherapy and regular follow-up were conducted through outpatient visits or telephone calls. Follow-up occurred every 3 months for the first year after surgery and every 6 months thereafter until death. Follow-up mainly involved imaging studies and measurement of tumor markers for the assessment of disease progression. The main observation index was OS, which was defined as the period from initial preoperative chemotherapy to the time of death from any cause. Disease-specific OS was defined as the period from initial preoperative neoadjuvant chemotherapy to the time of death due to progression of GC. The last follow-up date for this study was January 31, 2017.

In regard to the specimens obtained from surgery, the percentage of residual tumor cells, that is, GHR, within the lesion was recorded as 0–100%; 0% represents no necrosis, cellular or structural changes within the entire lesion, while 100% represents an entire lesion that was replaced by fibrous tissue with no viable tumor cells present (pathologic complete response, pCR).

### Statistical analysis

Patients were grouped according to different GHR criteria, and the clinical staging of the different groups before neoadjuvant chemotherapy was compared.

#### Univariate analysis of overall survival-related factors

For all 87 patients, we compared OS with respect to age, tumor differentiation, tumor location, pre-neoadjuvant chemotherapy TNM staging, postoperative pathological staging (ypTNM), Lauren classification, neural invasion, vascular invasion, and GHR to determine factors that affect the prognosis of GC patients. For the GHR subgroup, we grouped patients according to different GHR values (30%, 35%, 40%, 45%, 50%, 60%, 66.7%, and 90%) and performed a univariate analysis. A survival analysis was performed for 85 patients; 2 patients who died due to non-disease-related progression were not included in the analysis.

#### Multivariate analysis of overall survival-related factors

Cox regression analysis was applied to evaluate the 87 patients grouped according to different GHR values (40%, 45%, 50%, 60%, 66.7%, and 90%) with respect to gender, tumor differentiation, tumor location, pre-neoadjuvant chemotherapy TNM stage, postoperative pathological staging (ypTNM), Lauren classification, neural invasion, vascular invasion, and other relevant factors. We calculated and determined important independent risk factors that influenced the prognosis of patients with GC. The abovementioned multivariate survival analysis was performed for 85 patients, as 2 patients who died due to non-disease-related progression were not included.

SPSS statistical software 22.0 (version 22.0; SPSS Inc., Chicago, IL, USA) was used for all the statistical analyses. Age data are presented as medians. Numerical data are presented as percentages (*n*, %), and intergroup comparisons were performed using the Mann-Whitney test. Survival analysis was performed according to the Kaplan-Meier method, and intergroup comparisons were performed with the log-rank test. A multivariate analysis was performed using Cox regression analysis, and Spearman’s rank correlation or Pearson’s correlation analysis was used to evaluate correlations. The threshold for statistical significance was set at *p* < 0.05, and all *p* values are from two-tailed tests.

## Results

### General clinicopathological results

Of the 87 patients, 66 were male (75.9%) and 21 were female (24.1%). The median age was 56 years (Table 1). Overall, 3 to 4 courses of neoadjuvant chemotherapy were administered, and 67 (77.0%) patients received the Folfox regimen (see Table 1 for details). Postoperative pathological evaluation revealed different degrees of GHR for all the patients; 50%, 66.7%, and 90% GHR were observed in 50.6%, 34.5%, and 17.2% of the patients, respectively. Four of the patients achieved pathological complete response (4.6%). Seventy-nine patients continued to receive chemotherapy after surgery, and the median number of chemotherapy cycles was 5.

**Table 1:** Clinicopathological features of the 87 patients

Clinicopathological features	*n* = 87(%)
Age (median year, range)*	56 (47–65)
Sex, *n* (%)	
Male	66 (75.9)
Female	21 (24.1)
Tumor differentiation, *n* (%)	
Well-/median-differentiated	15 (17.2)
Poorly differentiated/mucinous or signet ring cell carcinoma	72 (82.8)
Tumor location, *n* (%)	
Upper body	16 (18.4)
Middle body	27 (31.0)
Lower body	41 (47.1)
Diffuse type	3 (3.4)
Pre-chemotherapy T stage, *n* (%)	
T0–2	4 (4.6)
T3–4	83 (95.4)
Pre-chemotherapy N stage, *n* (%)	
N−	18 (20.7)
N+	69 (79.3)
Pre-chemotherapy TNM, *n* (%)	
II	41 (47.1)
III	44 (50.6)
IV	2 (2.3)
Neoadjuvant chemotherapy regimen, *n* (%)	
FOLFOX	67 (77.0)
XELOX	15 (17.2)
SOX	4 (4.6)
Lauren classification, *n* (%)	
Intestinal	30 (34.5)
Diffuse	49 (56.3)
Mixed	8 (9.2)
Neural invasion, *n* (%)	
Yes	1 (1.1)
No	86 (98.9)
Vascular invasion, *n* (%)	
Yes	12 (13.8)
No	75 (86.2)
ypT stage, *n* (%)	
T0–1	9 (10.3)
T2–3	55 (63.2)
T4	23 (26.4)
ypN stage, *n* (%)	
N0	39 (44.8)
N1+N2	28 (32.2)
N3	20 (23.0)
ypTNM stage, *n* (%)	
0+I	16 (18.4)
II	40 (46.0)
III	31 (35.6)
GHR, *n* (%)	
≥ 50%	44 (50.6)
< 50%	43 (50.8)
GHR, *n* (%)	
≥ 2/3	30 (34.5)
< 2/3	57 (65.5)
GHR, *n* (%)	
≥ 90%	15 (17.2)
< 90%	72 (82.8)

Data are presented as a percentage (%). *GHR* graded histological regression

*Not normally distributed, presented as the median (M) and interquartile range

### Univariate and multivariate analyses of relevant factors for overall survival

The Mann-Whitney test revealed almost no significant difference in the pre-chemotherapy cTNM stage between the two groups with different GHR criteria (see Table 2 for details).

**Table 2:** Significance testing for the group of 87 patients before neoadjuvant chemotherapy

Pre-chemotherapy TNM stage					
GHR	*n* (%)	II	III	IV	*p* value*
≥ 45%	45	18 (40.0)	25 (55.6)	2 (4.4)	0.124
< 45%	42	23 (54.8)	19 (45.2)	0 (0.0)	
≥ 50%	44	17 (38.6)	25 (56.8)	2 (4.5)	0.078
< 50%	43	24 (55.8)	19 (44.2)	0 (0.0)	
≥ 2/3	30	9 (30.0)	20 (66.7)	1 (3.3)	0.021
< 2/3	57	32 (56.1)	24 (42.1)	1 (1.8)	
≥ 90%	15	4 (26.7)	10 (66.7)	1 (6.7)	0.062
< 90%	72	37 (51.4)	34 (47.2)	1 (1.4)	

Data are presented as numbers (%).*GHR* graded histological regression

*Mann-Whitney test

Univariate and multivariate survival analyses were performed to detect overall survival-related risk factors among 87 gastric cancer patients.

**Fig. 1:**
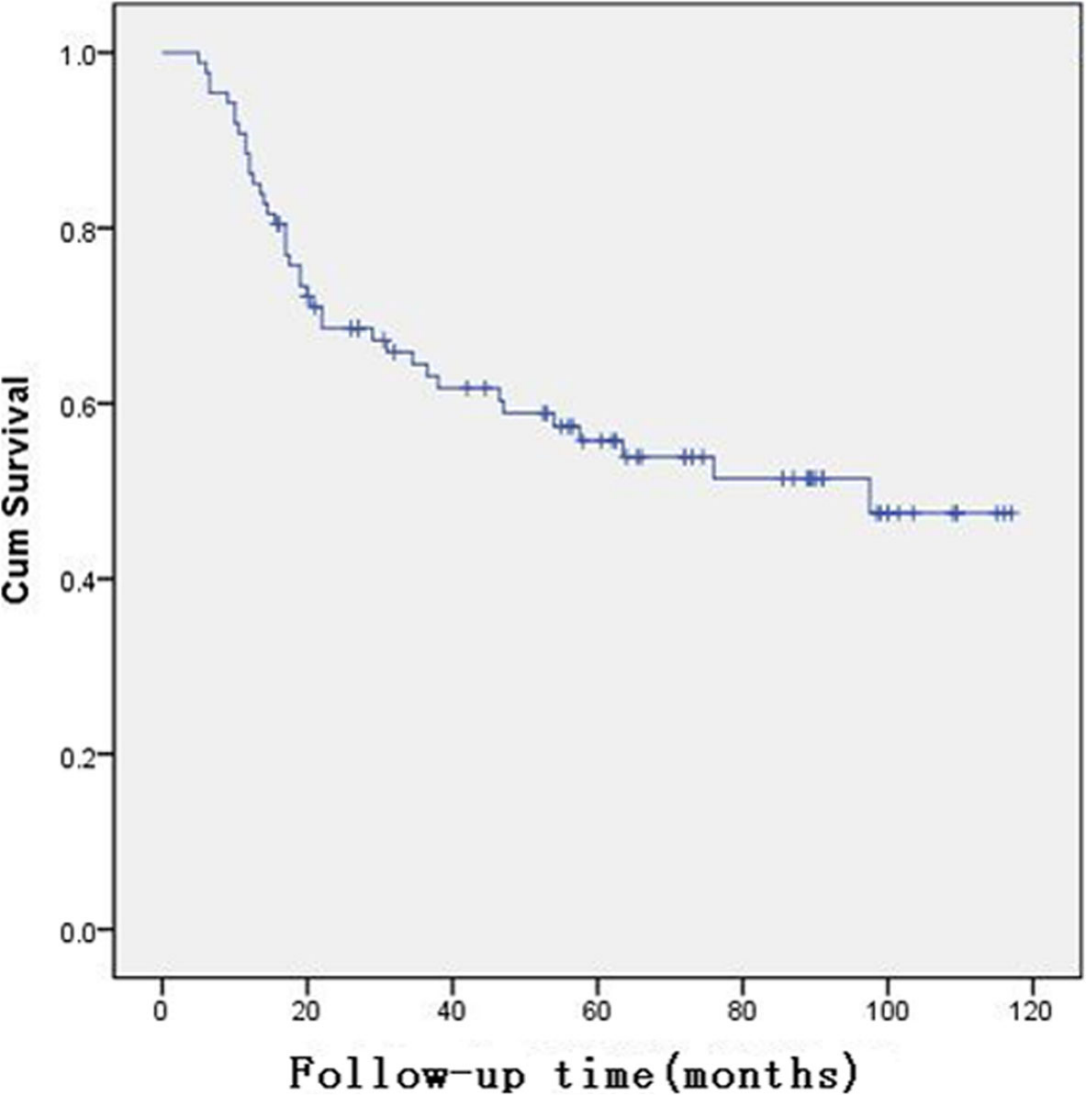
Overall survival curve for the 87 patients

All 87 patients who underwent surgery completed follow-up, with a median follow-up time of 45 months (range 5 to 117 months). Overall, 41 patients (47.1%) died, 39 (44.8%) of whom died due to recurrence of GC, whereas 2 died from other causes. The median OS time was 97.5 months (Fig. 1), and the 1-year, 2-year, 3-year, 5-year, and 7-year survival rates were 86.2% (95% CI 78.9–93.5%), 68.5% (95% CI 58.7%–78.3%), 64.5% (95% CI 54.3–74.7%), 55.7% (95% CI 44.7–66.7%), and 51.4% (95% CI 39.6–63.2%), respectively.

**Fig. 2:**
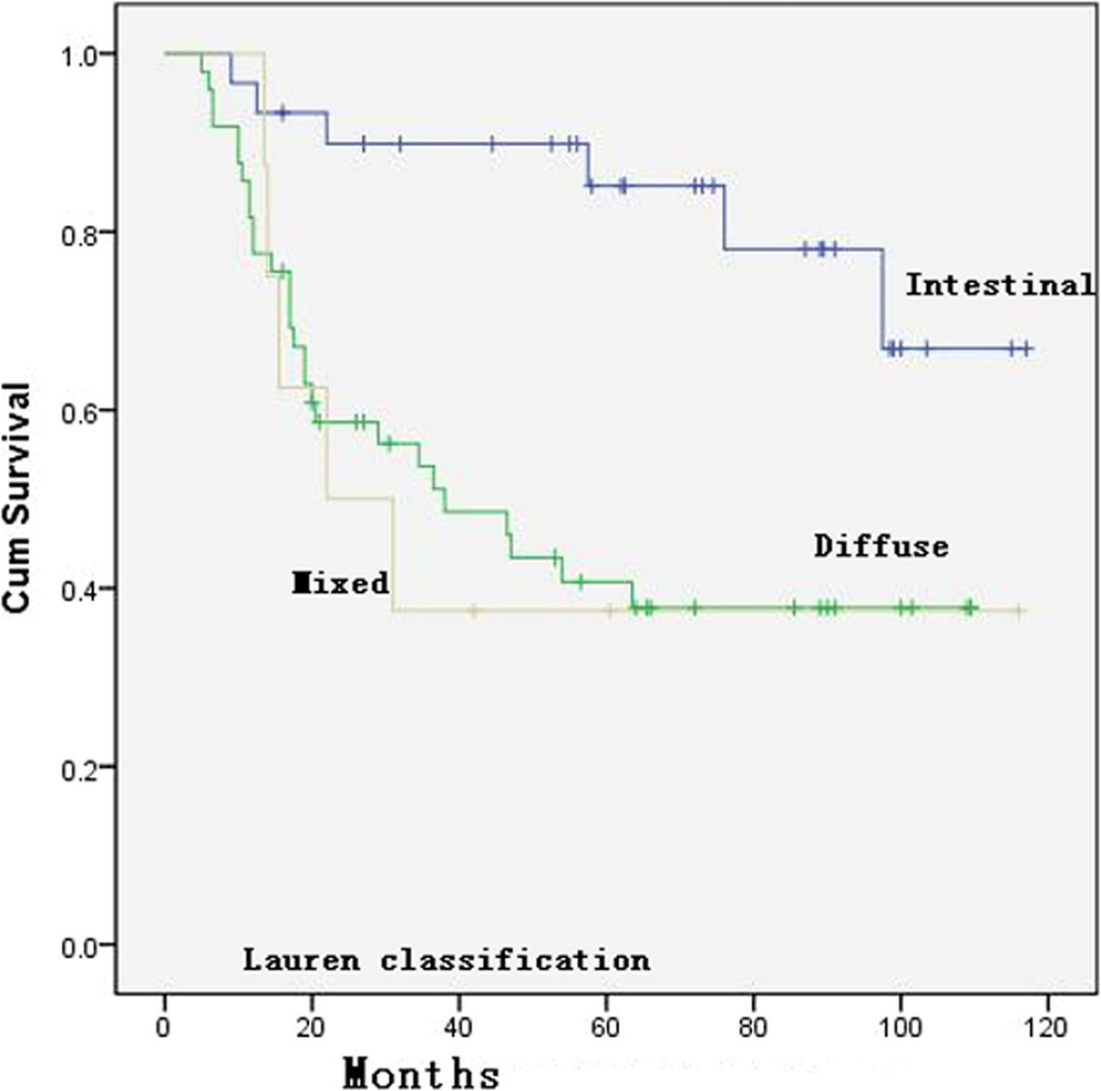
Overall survival curve for the 87 patients with different Lauren classification

**Fig. 3:**
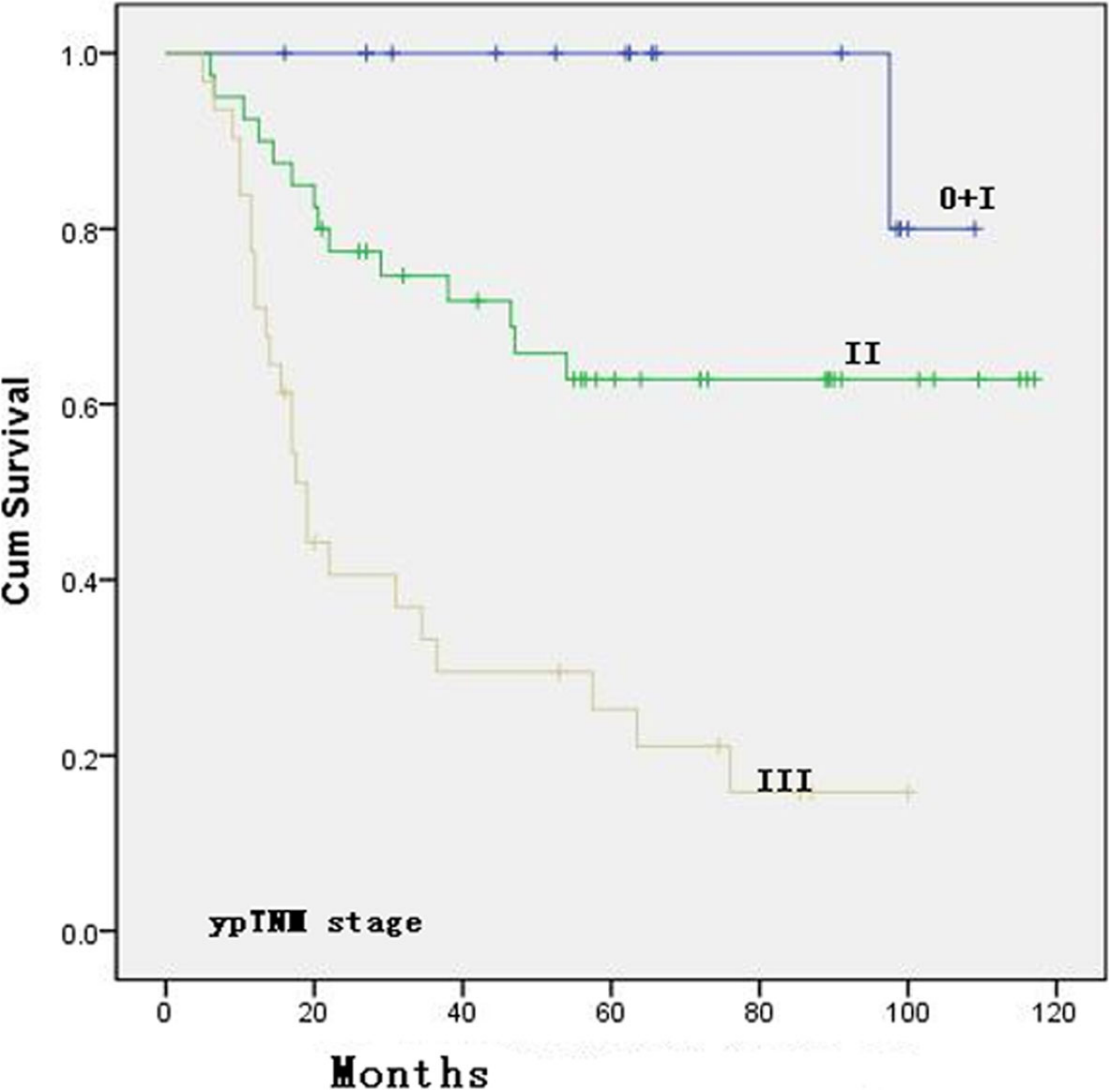
Overall survival curve for the 87 patients with different ypTNM stages

The univariate analysis showed that the Lauren classification (*p* = 0.002, Fig. 2) and ypTNM stage (*p* = 0.001, Fig. 3) were significantly correlated with OS. When the patients were grouped and compared according to different GHR rates (90%, 66.7%, 50%, 45%, 40%, and 35%), the GHR of the primary lesion was correlated with survival when GHR was 50%, 66.7%, or 90% (see Table 3). The multivariate analysis demonstrated that ypTNM (II/III) stage was significantly associated with OS compared with ypTNM (0+I) stage [HR = 3.553, 95% CI 1.886–6.617; HR = 3.576, 95% CI 1.908–6.703, respectively] and that the Lauren classification of diffuse type was also an independent risk factor for OS compared with the intestinal type (HR = 3.843, 95% CI 1.443–10.237) (details in Table 3).

**Table 3:** Survival-related prognostic factors for the 87 patients

Variable	Univariate analysis			Multivariate analysis		
	*n* = 87(%)	Median survival (months)	*p* value	HR	95% CI	*p* value
Sex			0.542			
Male	66 (75.9)	76.0		1 (Ref)		
Female	21 (24.1)	–		1.552	0.637–3.777	0.333
Tumor location			0.009			0.104
Upper body	16 (18.4)	57.5		1 (Ref)	–	–
Middle body	27 (31.0)	–		0.347	0.132–0.915	0.032
Lower body	41 (47.1)	–		0.532	0.216–1.310	0.17
Diffuse type	3 (3.4)	15.0		1.193	0.254–5.597	0.823
Preoperative TNM stage			0.361	1.063	0.521–2.168	0.867
II	41 (47.1)	57.5		1 (Ref)		
III+IV	46 (52.9)	97.5				
Lauren classification			0.002			0.025
Intestinal	30 (34.5)	–				
Diffuse	49 (56.3)	38.0		3.843	1.443–10.237	0.007
Mixed	8 (9.2)	22.0		2.624	0.698–9.863	0.153
Vascular invasion			0.342			
Yes	12 (13.8)	46.5		0.908	0.370–2.232	0.834
No	75 (86.2)	–		1 (Ref)		
Tumor differentiation			0.269			
Well-/median differentiated	15 (17.2)	76.0				
Poorly differentiated/mucinous or signet ring cell carcinoma	72 (82.8)	63.0		1.429	0.455–4.486	0.541
ypTNM			0.001			< 0.001
0+I	16 (18.4)	–		1 (Ref)		
II	40 (46.0)	–		3.533	1.886–6.617	< 0.001
III	31 (35.6)	19.0		3.576	1.908–6.703	< 0.001
GHR			0.022			
≥ 50%	44 (50.6)	–		1 (Ref)		
< 50%	43 (49.4)	34.5		1.171	0.653–2.764	0.689
GHR			0.013			
≥ 2/3	30 (34.5)	–		1 (Ref)		
< 2/3	57 (65.5)	46.5		1.654	0.680–4.024	0.267
GHR			0.028			
≥ 90%	15 (17.2)	–		1 (Ref)		
< 90%	72 (82.8)	54.0		0.998	0.254–3.917	0.998

The results of multivariate analyses are based on the criteria of GHR≥50%/< 50%. Data are presented as number (%). *OR* odds ratio, *CI* confidence interval, *GHR* graded histological regression, *Ref* reference, *HR* hazard ratio

The follow-up time of the 85 patients (excluding 2 patients with non-GC-related death) ranged from 5 to 117 months, and the median disease-specific OS was 73 months. The 1-, 2-, 3-, 5-, and 7-year survival rates were 87.1% (95% CI 78.4–92.3%), 69.0% (95% CI 56.7–77.8%), 63.4% (95% CI 52.2–71.6%), 54.0% (95% CI 43.1–62.7%), and 51.3% (95% CI 34.6–62.3%), respectively. The univariate and multivariate analyses of the 85 patients are consistent with the aforementioned results of the 87 patients.

## Discussion

Many large-scale clinical trials and meta-analyses have indicated that patients with advanced GC who received perioperative chemotherapy including neoadjuvant chemotherapy had a higher rate of R0 resection and longer disease-free survival and OS than patients who underwent surgery alone [3, 4, 19, 20]. In this study, the median survival of 87 patients with advanced GC was 97.5 months, while the 5-year OS rate was 54%. Although a control group of patients who underwent surgery alone was not established in this study, the results of our study were similar to those of other analogous and comparative studies [13] and could report the value of perioperative chemotherapy to a certain degree. Ultimately, patients who directly benefit from neoadjuvant chemotherapy (chemotherapy effective) exhibit long-term survival, whereas those for whom neoadjuvant chemotherapy is ineffective have the opportunity to be treated with potentially effective drugs that will benefit their overall treatment plan and help them achieve an improved prognosis and prolonged OS [2]. Thus, methods and standards for determining the efficacy of neoadjuvant chemotherapy (namely, distinguishing between patients who respond differently to chemotherapy regimens) need to be developed to achieve the goal of an overall treatment program.

At present, two primary methods are used to judge the efficacy of neoadjuvant chemotherapy in GC: imaging and pathology. Imaging evaluations mainly refer to the solid tumor reaction standard, as achieved by spiral CT and other techniques, to determine efficacy. However, such an evaluation is associated with many issues, including high demand regarding the experience of the imaging physician and the poor correlation between results and prognosis [21, 22]. Several studies have shown that the accuracy of enhanced CT after neoadjuvant chemotherapy in T staging and N staging is only 57% and 37%, respectively, mainly because it is difficult to distinguish between tissue fibrosis after chemotherapy and the tumor itself using CT [23]. Our previous study also found that the consistency between imaging after neoadjuvant chemotherapy and postoperative pathological testing is not high, which demonstrates that the efficacy, as determined by imaging, compared with inefficacy failed to show a longer survival time (*p* = 0.438) [24].

Pathological response, mainly through GHR, is commonly used for assessment in China and elsewhere to reflect efficacy. Many studies have reported that pathological assessment provides a good indication of patient prognosis [15–17, 24]. Currently, widely adopted GHR criteria include the JCGC pathological evaluation criteria and Becker’s histological tumor regression grade [15, 16]. However, the percentage of efficacy using the Becker criteria is low [17, 25] and was only 22.4% in our previous study [24], which is similar to the result in the present study (17.2%). Kurokawa et al. and Tsuburaya et al. used GHR criteria of the JCGC and reported efficacy rates of 34% [18] and 29% [13], respectively. In our previous study, the efficacy rate was 29.9% [24], which is similar to the findings of the current study (34.5%). For 20–30% efficacy under such criteria, researchers must consider whether the majority of patients with neoadjuvant chemotherapy need to change their postoperative chemotherapy regimen or whether the GHR evaluation criteria should be adjusted.

**Fig. 4:**
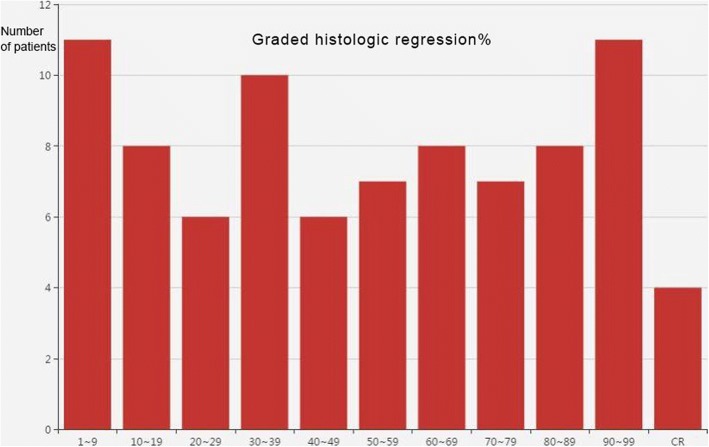
Distribution of GHR for the 87 patients. Graded histological regression (GHR)

**Fig. 5:**
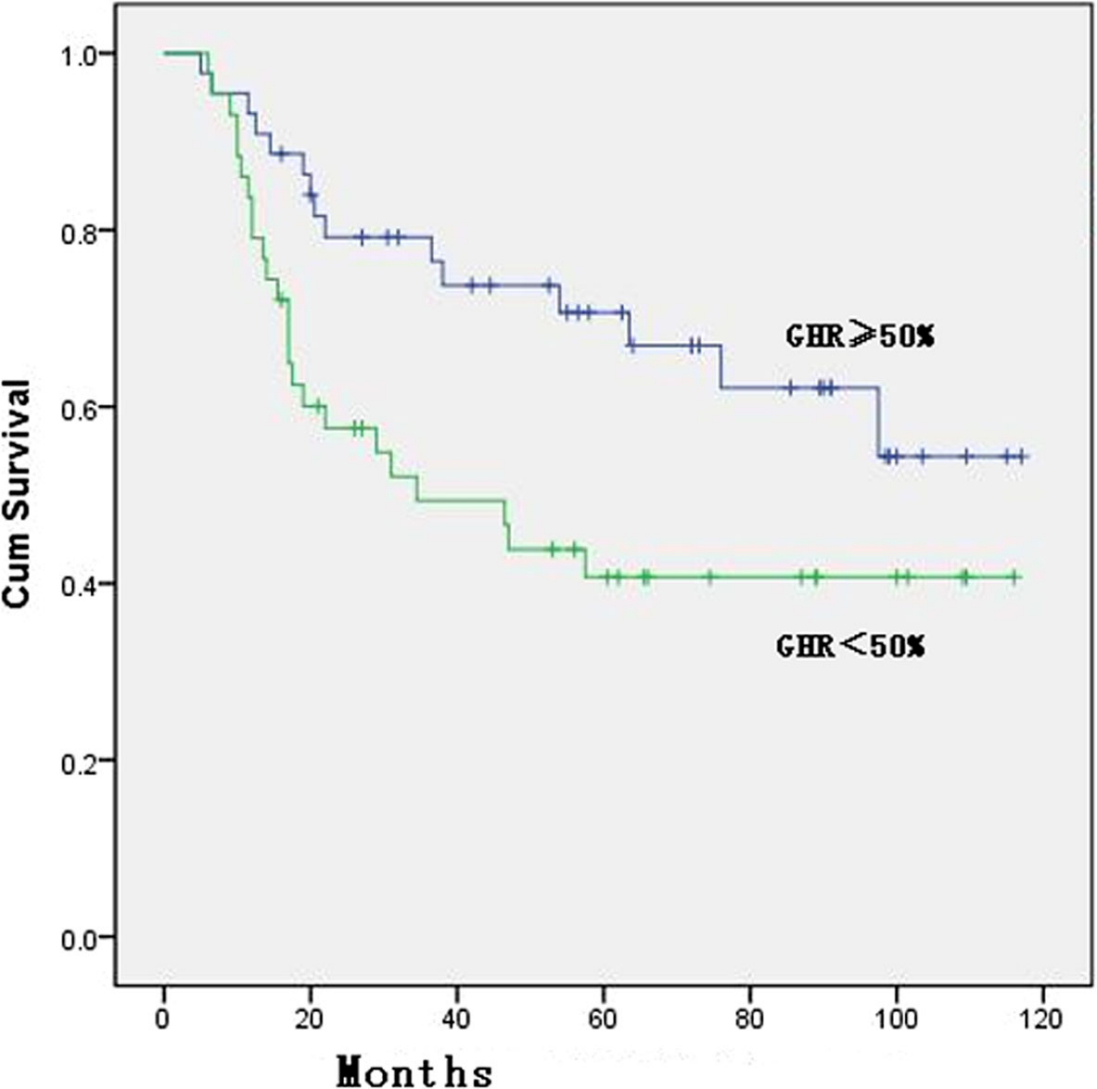
Overall survival curve of the GHR ≥ 50%/< 50% for the 87 patients. Graded histological regression (GHR)

In this study, a univariate analysis of either all 87 patients or 85 patients after the exclusion of 2 who died of non-disease progression revealed that tumor location (*p* = 0.009, Fig. 4), Lauren classification (*p* = 0.002, Fig. 2), and ypTNM stage were highly significantly correlated with OS, which was consistent with what was reported in previous studies [24, 26, 27]. When patients were grouped according to different GHR rates, the median survival time of the effective group was significantly longer than that of the ineffective group (*p* = 0.028, 0.013, respectively) according to the Becker criteria and the JCGC criteria. Compared with a GHR < 50%, GHR ≥ 50% showed a significant survival benefit (no benefit vs 34.5 months, *p* = 0.022), which was consistent with what was shown in previous studies [15, 24, 25, 28]. In addition, Mansour et al. [29] found a significant difference in the 3-year survival between patients with a GHR ≥ 50% and a GHR < 50% (69% vs. 44%, *p* = 0.01). These results suggest that using a GHR ≥ 50% and more “stringent” standards, the overall median survival time and survival rate for the effective group are significantly better than those of the control group and that GHR is an indicator of survival and prognosis. It is worth noting that when a GHR ≥ 50%/< 50% is used as a pathological criterion, the efficacy rate was 50.6% (Fig. 5; in our previous study, this rate was 49.2%. The rates reported by Ferri et al. and two other studies were 49%, 45.6%, and 46.4% [17, 25, 28], which were higher than the 20–30% rate based on the Becker and JCGC criteria.

Our multivariate analysis results did not show a significant correlation between GHR and OS. In contrast, the Lauren classification and ypTNM stage were significantly associated with OS, which again verifies our previous findings [24]. Studies by Sylvie et al. and Viani et al. also showed that the Lauren classification is an independent prognostic factor that affects the survival of patients with advanced GC [26, 27]. To some degree, the Lauren classification is related to tumor biological behavior, which may explain its prognostic value [30]. Diffuse GC often occurs in younger individuals and often presents with early regional lymph node metastasis and distant metastasis [31–33]. Both the univariate and multivariate analyses showed that ypTNM stage is an important independent prognostic factor, which again verified our previous results. Schmidt, Davies, Lowy, and others have also reached the same conclusion [22, 24, 34, 35]. In our multivariate analysis, grouping was performed using the GHR ≥ 50%/< 50% criterion or the more stringent Becker and JCGC criteria, which was followed by regression analysis to calculate the survival time. The results revealed no significant difference between the effective and ineffective groups (*p* values were 0.689, 0.998, 0.267; see Table 3). The studies by Fujitani and Schmidt et al. also support this finding [22, 36], which suggests that the degree of tumor necrosis after chemotherapy does not independently affect patient survival and that it only affects survival significantly when chemotherapy-induced tumor necrosis significantly induces downstaging. Thus, patients who have significant tumor necrosis confirmed by postoperative pathology and a lower ypTNM stage may directly benefit from neoadjuvant chemotherapy and achieve long-term survival.

Although GHR was not an independent prognostic factor for survival in our multivariate analysis, we recommend using a GHR ≥ 50%/< 50% as the primary pathologic criterion for patients with advanced GC after they receive neoadjuvant chemotherapy. This recommendation is in accordance with our univariate analysis and can be used to determine the appropriate postoperative adjuvant chemotherapy regimen. The reasons are described as follows: (1) According to our univariate survival analysis, the effective group showed a significantly longer survival time than the control group using the GHR ≥ 50% criterion or the more “stringent” Becker and JCGC criteria; however, the stricter criteria failed to exhibit independent effects on survival time in the multivariate analysis. In contrast, the criteria for GHR below 50% failed to show a significant effect on survival in the univariate analysis. (2) Many studies have shown that the overall clinical efficacy of common chemotherapy drugs is approximately 50%; moreover, the proportion of efficacy determined by the GHR ≥ 50%/< 50% criterion was approximately 50%. Both values are close to each other and are therefore more easily accepted and promoted clinically. (3) The nearly 70–80% of “ineffective” patients who require a change in their postoperative chemotherapy regimen will become strong clinical burdens if neoadjuvant chemotherapy is evaluated according to the currently used Becker and JCGC criteria. Therefore, we propose distinguishing GC patients after neoadjuvant chemotherapy by the GHR ≥ 50%/< 50% criterion, which is practical and feasible. For those with a GHR rate of less than 50%, continuation of the preoperative chemotherapy regimen as a postoperative adjuvant chemotherapy regimen is not recommended.

## Conclusions

In summary, the Lauren classification and ypTNM stage after neoadjuvant chemotherapy are independent prognostic factors for advanced gastric cancer. GHR ≥ 50%/< 50% can be used as the primary criterion to evaluate the curative effects of neoadjuvant chemotherapy in advanced GC and to guide the selection of postoperative adjuvant chemotherapy regimens.

As this report describes a single-center, small-scale study without control groups, the sample size should be expanded in a future study to confirm these findings.

## Abbreviations

CI: Confidence interval
GC: Gastric cancer
GHR: Graded histological regression
HR: Hazard ratio
OR: Odds ratio
OS: Overall survival
